# Endotype and Sample Site-Related Periostin Upregulation in Chronic Rhinosinusitis with Nasal Polyposis: A Systematic Review

**DOI:** 10.1055/a-2804-5314

**Published:** 2026-11-04

**Authors:** Omar A. Alanzi, Sarah A. Almansour, Mohammad F. Aldajani, Moath A. Alfaleh, Maria G. Riga

**Affiliations:** 1 Department of Otorhinolaryngology-Head & Neck Surgery, Dammam Medical Complex, DammamKingdom of Saudi Arabia.

**Keywords:** periostin, chronic rhinosinusitis with nasal polyposis, biomarker, eosinophilic

## Abstract

**Introduction:**

The present literature lacks an endotype- or anatomical site-targeted synthesis of the studies advocating for periostin upregulation in nasal polyps (NPs).

**Objectives:**

Differences in periostin levels between NP samples from different CRSwNP endotypes, NP and normal controls, NP and nonpolypoidal mucosal samples (MSs) within chronic rhinosinusitis with nasal polyposis (CRSwNP) patients, and MS from CRSwNP patients and controls were considered as primary outcomes.

**Data synthesis:**

Eosinophilic NP (eNP) samples exhibited significantly higher periostin levels than non-eNP, while non-eNP samples were found to be comparable to controls. Comparisons between NP and MS of patients with CRSwNP attributed significantly higher periostin mRNA and protein levels in the former, while the MS of patients with CRSwNP were comparable to controls and CRSsNP. A strong correlation between mRNA and protein periostin measurements was verified.

**Conclusion:**

Periostin is highly associated with eosinophilic CRSwNP and significantly upregulated in mature eNP rather than nonpolypoidal mucosa.

## Introduction

Periostin is an extracellular matrix protein, which promotes cell adhesion, autocrine activation and collagen fibrinogenesis in response to injury and inflammation. 1 2 3 Over the past decade, the upregulation of periostin in nasal polyp (NP) tissue samples of patients with chronic rhinosinusitis (CRS) with nasal polyposis (CRSwNP) compared with controls has triggered broad research on the possible pathogenetic role of periostin and its potential use as a biomarker/therapeutical target in the treatment of CRSwNP.

The present systematic review targets a better insight into the role of periostin in the pathophysiology of CRSwNP, by determining the details of periostin upregulation regarding different CRSwNP endotypes, different sites of the nasal mucosa, and CRS patients without CRSsNP. The second question will be summarized through the differences between mature NP tissue samples and nonpolypoidal mucosal samples (MSs) collected from different adjacent anatomical sites within the same CRSwNP patients, in comparison with normal controls. The clinical usefulness of periostin as a potential biomarker, as well as variances in the methodology used by different research groups, were also evaluated and discussed.

## Methods

This review adhered to the guidelines of the Preferred Reporting Items of Systematic Reviews and Meta-Analysis (PRISMA) statement. 4 5 Prospective and retrospective studies referring to periostin levels in NP, MS, nasal secretions or serum were all eligible for inclusion.

A review of the present literature was conducted in the PubMed CENTRAL, and Openmd.com databases. The pubmed search was conducted for human studies only and time-limited for the period January 2008 to June 2025. With these limits applied, the search terms periostin and sinusitis attributed 75 results and the terms periostin and nasal polyp attributed 67 results. In Openmd.com the same search terms produced 39 and 48 results respectively. No relevant/similar reviews appeared from the search in CENTRAL database. Articles written in Chinese were excluded.

The procedure is presented as a flowchart in Fig. 1 . The study inclusion criteria have been described in detail in a previous publication. 6 7 8

**Fig. 1:**
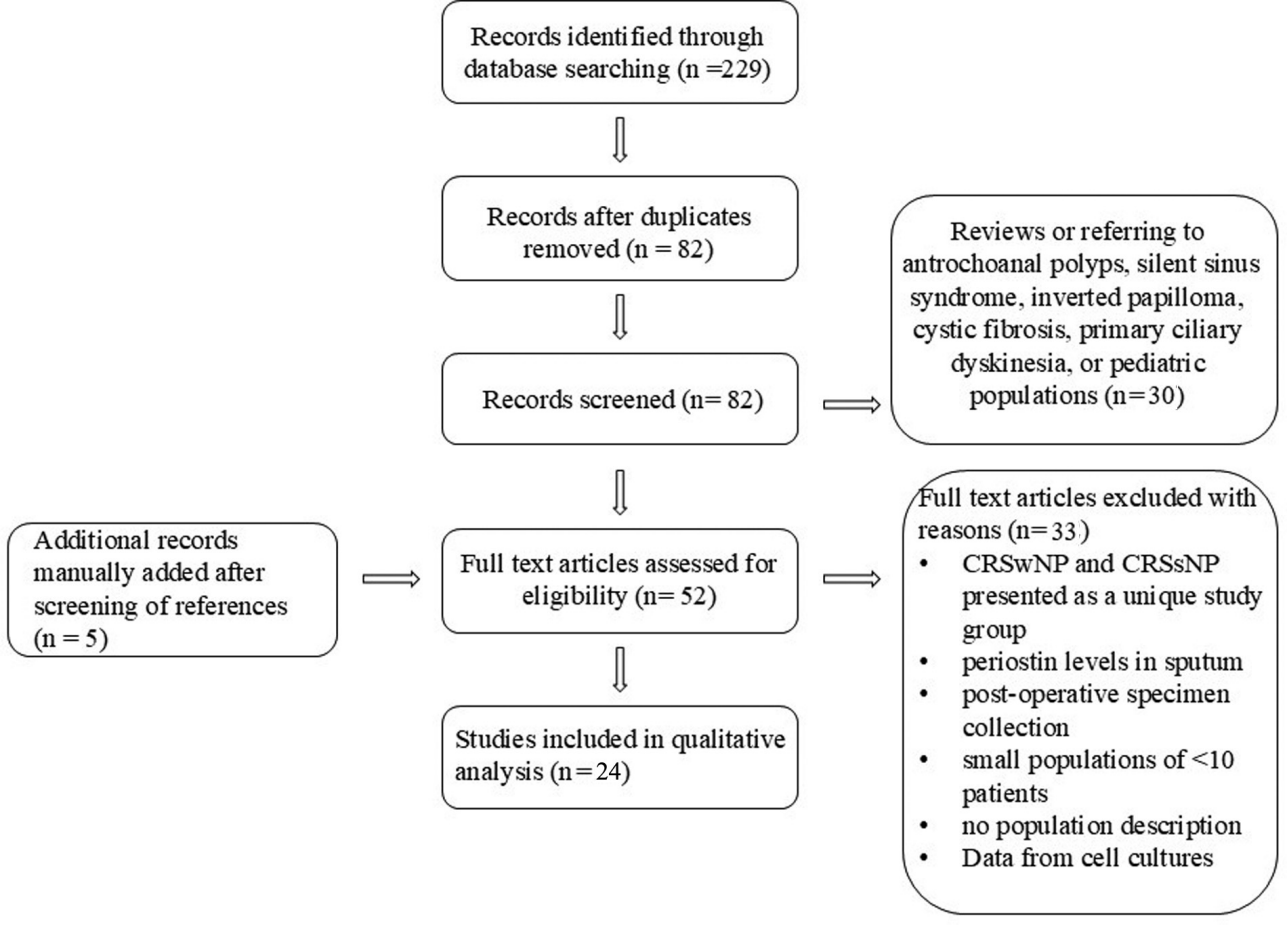
Study selection flowchart.

Two investigators (OA and MR) independently decided on the study eligibility and extracted data from full text manuscripts.

We considered as primary outcomes the differences in periostin mRNA and protein levels between CRSwNP patients and normal controls. Both NP and MS (such as uncinate process [UP], inferior/middle turbinate, or other non-polypoidal mucosal tissue samples) taken from CRSwNP patients were compared with controls. We also considered as primary outcomes the differences in periostin levels between NP and MS within the same CRSwNP patients and among different CRSwNP endotypes. Secondary outcomes included the accordance between mRNA and protein measurements, the conformity between different laboratory methods of measuring periostin and the differences between CRSwNP and CRSsNP patients.

The Quality Assessment of Diagnostic Accuracy Studies (QUADAS-2) tool was used to evaluate the included studies. 5 The tool consists of 4 domains; 1) patient selection, 2) index test (in this study periostin measurements), 3) reference standard (in this study CRSwNP diagnosis, including any endotype assessment), 4) flow and timing between the diagnosis of CRSwNP and measurements of periostin levels.

Βased on the bias factors determined in a previous review, 8 a high risk for selection bias was attributed if ≥3 of the following bias factors were unknown or significantly different among study groups; asthma, non-steroidal anti-inflammatory drug exacerbated respiratory disease (NERD), atopy, disease severity (assessed with polyp size, clinical or radiological severity), and local or systemic steroid treatment ( Table 1 ).

**Table 1:** Patient selection bias

N	Study	Asthma	NERD	Atopy	Severity	Glyco-corticosteroids	Other interventions	Eosinophilic endotype
**Studies on tissue samples**								
1	Stankovic et al., 2008 20	NS (CRSwNP vs. CLS)	Excl	?	Bilateral NP, frontal and/or sphenoid incl	Oral excl	?	No
2	Platt et al., 2011 30	CRSwNP > CLS	Excl	?	Pansinusitis & NP grade 3	Oral excl	?	No
3	Ishida et al., 2012 9	CRSwNP > CLS	Subgroup	Study subgroup	CT ± SD 3.5 ± 0.6	Oral excl	No immune-modulating drugs	No
4	Kim et al., 2015 10	NS (E vs. NE)	?	IgE Ab against 6 mixtures of common aeroallergens> 3.51 kU/L; E > NE	L-M [15(10-20) vs. 16(10-22)], E/M ratio, olfactory cleft opacification	Syst & IN excl	No antibiotics, antihistamines or Immune-modulating drugs	Yes
5	Seshardi et al., 2015 33	CRSwNP > CLS	?	?; CRSwNP > CLS	?	?	?	No
6	Shiono et al., 2015 21	Diffuse > superficial periostin	None	NS	?	?	?	Yes
7	Wang et al., 2015 22	NS	NS	Skin prick test +; NS	mean (range) L-M: 14 (2-24) NP grade: 1.5 (0.5-3)	Syst & IN excl	No antibiotics	Yes
8	Gevaert et al., 2017 28	NS (CRSwNP vs. CLS)	?	NS (CRSwNP vs. CLS)	?	Syst & IN excl	?	Yes
9	Kim et al., 2017 23	NS	Excl	IgEAb for 6 mixtures of aeroallergens and tissue total IgE; NS	?	Syst & IN excl	No antibiotics, antihistamines or immune-modulating drugs	Yes
10	Xu et al., 2017 11	NS	?	?	?	Syst & IN excl	?	Yes
11	Wei et al., 2018 27	CRSwNP > CLS Among CRSwNP E asthma = 9 and NE asthma = 5	NS	Skin prick tests CRSwNP > CLS	(mean ± SD) L-M: 15.8 ± 10 NP grade: 2 ± 0.8	?	?	Yes
12	Wu et al., 2018 29	E-CRSwNP > CLS	NS	Skin prick tests NS	(mean ± SD) L-M: 15.7 ± 4.3 L-K: 6.7 ± 2.6	Syst & IN excl	No antibiotics	Yes
13	Wu et al., 2018 12	CRSwNP > CLS	NS	NS	(mean ± SD) L-M: 15.7 ± 4.3 L-K: 6.7 ± 2.6	Syst & IN excl	No antibiotics	Yes
14	Nakayama et al., 2019 13	E > NE	NS	?	L-M, NP grade mean ± SD by groups	Oral excl	No antibiotics	Yes
16	Du et al., 2021 15	NS	NS	CRSwNP > CLS	mean (range) L-M: 14 (2-24)	Syst & IN excl	No antibiotics	Yes
17	Yilmaz et al., 2022 19	CRSwNP > CLS	CRSwNP > CLS	CRSwNP > CLS Diagnosis of allergy only with history	(mean ± SD) L-M: 19.9 ± 4	Syst & IN excl	?	Yes
18	Zhang et al., 2022 16	NS	?	NS	(mean ± SD) **Normosmic:** L-M: 9.5 ± 6.5 L-K: 4.4 ± 3.7 **Hyposmic:** L-M: 15.6 ± 7 L-K: 7.2 ± 3.3	Oral excl	?	Yes
19	Danielides et al., 2025 17	Investigated as bias factor	?	?	?	Syst & IN excl	Smoking tested as bias factor	No
20	Maharani et al., 2025 18	NS, 1/30pts	Excl	excl	(mean ± SD) L-K: 1.4 ± 0.8 L-M: 13.2 ± 6.3	Syst & IN excl	No antibiotics	Yes
21	Suzaki et al., 2025 25	CRSwNP > CLS E > NE	CRSwNP > CLS E > NE	?	?	Syst excl		Yes
**Studies on serum and/or nasal secretions**								
22	Asano et al., 2017 31	All asthmatic	NS	IgEAb for 8 common aeroallergens; NS	L-M for each pt in dot plots	Oral excl	No immune-modulating drugs	Yes
23	Maxfield et al., 2017 32	CRSwNP > CLS	CRSwNP > CLS	?	?	Multivariate regression analysis; NS	?	No
24	Xu et al., 2017 26	NS	?	?	?	Syst & IN excl	excl	Yes
25	Wu et al., 2021 14	Uncontrolled > controlled CRSwNP	Excl	Uncontrolled > controlled CRSwNP	?	Syst & IN excl	No antibiotics or immune-modulating drugs	Yes

**Abbreviations:** ?, not reported; Ab, antibodies; CLS, controls; CT, computed tomography; E/M ratio, ethmoid/maxillary L-M score ratio; excl: excluded from study population; IN, intranasal; L-K, Lund Kennedy score; L-M, Lund McKay score; NS, nonsignificant between study groups; NSAID, nonsteroidal anti-inflammatory drugs; pts, patients; syst, systemic. **Notes:** > indicated significant difference between study groups.

With respect to the interpretation of periostin levels (index test), low risk of bias was attributed if comparisons regarded samples of the same nature (serum, nasal secretions, MS or NP tissue) and unclear risk if the tests compared samples of different nature, such as NP versus MS tissues taken from different study groups. To evaluate any risk of bias regarding CRSwNP as a reference standard, we abstracted data on any analysis investigating the underlying inflammation pattern (eosinophilic, immunoglobulin [IgE] or interleukin 5 [IL-5] related), as shown in Table 1 . In case of lack of any such information, the study was considered of high risk of bias as far as CRSwNP diagnosis is concerned and classified as raising high concerns regarding the applicability of the results in CRSwNP populations.

The studies were divided into subgroups, according to the comparisons they made: 1) NP versus MS from healthy controls; CRSwNP, or CRSsNP patients; 2) MS from CRSwNP patients versus MS from controls; 3) NP from various CRSwNP endotypes or phenotypes with one another or controls,;4) serum; and 5) nasal secretions samples. Within individual studiesm, statistical significance was attributed to two-sided *p* < 0.05.

The prevalence of different CRSwNP endotypes may vary considerably among populations. Therefore, the lack of any investigation on the underlying inflammation patterns was classified as raising high concerns regarding reference standard applicability. To assess further risk of bias regarding reference standard and index text selections across studies, data was extracted on the methods used for the diagnosis of eosinophilic CRSwNP (E-CRSwNP) and the laboratory tests used to measure periostin levels ( Table 2 ). Data on the potential use of periostin as a biomarker were pooled.

**Table 2:** Summary of methods

N	Study	Population and Specimen	Consecutive or random	Study design	Eosinophilic diagnosis CRSwNP subgroups	Index test
**Comparisons between tissue samples**						
1	Stankovic et al., 2008 20	CRSwNP (NP) = 10 NERD (NP) = 10 CLS (IT, ES) = 10	?	Prospective case-control	CRSwNP vs NERD No eosinophil reference	IHC Microarray RNA RT-PCR
2	Platt et al., 2011 30	CRSwNP (NP) = 10 CRSwNP (IT, MT, S, LW) = 10 CLS (IT, MT, ES, S, LW) = 10	?	Prospective case-control	No eosinophil reference	RT-qPCR
3	Ishida et al., 2012 9	CRSwNP (NP) = 19 NERD (NP) = 10 CLS (IT) = 7	?	Prospective case-control	CRSwNP vs NERD No eosinophil reference	IHC qRT-PCR Western blotting
4	Kim et al., 2015 10	E (NP) = 36 NE (NP) = 45	?	Retrospective case-control	E if eosinophils> 10% of the inflammatory cell population	IHC qRT-PCR
5	Seshardi et al., 2015 33	CRSwNP (UP) = 23 CLS (UP) = 18	?	Prospective case-control	No eosinophil reference	IHC Semi qRT-PCR
6	Shiono et al., 2015 21	CRSwNP (NP) = 28	?	Retrospective	Diffuse (n = 20) vs superficial (n = 8) periostin expression in NP Shiono et al criteria for E	IHC
7	Wang et al., 2015 22	CRSwNP (NP) = 37 CLS (IT) = 15 CRSwNP (IT, ES) = 14	?	Prospective case-control	IL-5 low vs IL-5 high in NP tissue ECP, tissue and blood eosinophils in analysis	IHC ELISA RT-PCR
8	Gevaert et al., 2017 28	CLS (IT) = 5 CRSwNP (NP) = 15	?	Prospective case-control	ECP, tissue eosinophils in analysis	ELISA
9	Kim et al., 2017 23	CLS (UP) = 22 CRSwNP (UP) = 68 CRSwNP (NP) = 67 CRSsNP (UP) = 62 NE-(NP) = 34 E-(NP) = 38	?	Prospective case-control	E if eosinophils> 10% of the inflammatory cell population.	Histological analysis qRT-PCR
10	Xu et al., 2017 11	NE-(NP) = 36 E-(NP) = 30 CLS (MT) = 12	?	Prospective case-control	E if eosinophils> 10% of the inflammatory cell population	IHC qRT-PCR Western blotting
11	Wei et al., 2018 27	CLS (UP) = 25 CRSwNP (NP) = 63 NE(NP) = 16 E(NP) = 18	Random	Prospective case-control	E if > 7 eosinophilic cells/HPF (400 × )	IHC qRT-PCR Western blotting ELISA
12	Wu et al., 2018 12	CLS (IT) = 10 CRSwNP (NP) = 10	?	Prospective case-control	E if eosinophils per HPF > 10	Aptamer-based proteomic array and whole transcriptomic analysis
13	Wu et al., 2018 29	CLS (IT) = 10 E-CRSwNP (NP) = 10	?	Prospective case-control	E if eosinophils per HPF > 10	IHC Proteomic analysis Whole Transcriptomic Analysis
15	Nakayama et al., 2019 13	NE-(NP) = 9 E-(NP) = 42 CLS (UP) = 13	Consecutive	Prospective cross-sectional	E as per JESREC	qRT-PCR Luminex 200 for protein concentration
16	Du et al., 2021 15	CRSwNP (NP) = 37 CRSsNP (ES) = 14 CLS (IT) = 15	?	Prospective case-control	No eosinophil association with periostin seek	Immunoassay
17	Yilmaz et al., 2022 19	CRSwNP (NP) = 26 CLS (IT) = 30	?	Prospective case-control	E if eosinophils per HPF > 10	IHC Sandwich ELISA
18	Zhang et al., 2022 16	Preop normosmia (NP) = 25 Preop hypoanosmia (NP) = 60	?	Prospective cross-sectional	T&T olfactometer for olfactory function assessment Tissue eosinophils in analysis	IHC ELISA
19	Danielides et al., 2025 17	CRSwNP (NP) = 36; 13/36 asthmatic CLS (?) = 12	Consecutive	Prospective case-control	No eosinophil reference Subgroups according to asthma and smoking	ELISA RT-PCR Western blotting
20	Maharani et al., 2025 18	CRSwNP(NP, UP) = 30 CLS (IT) = 8	Consecutive	Prospective cross-sectional	Association with ECP Dominant biomarker	ELISA
21	Suzaki et al., 2025 25	CLS (UP) = 12 CRSwNP NE (NP) = 16 CRSwNP E (NP) = 14	?	Prospective case-control	E as per JESREC	IHC, ISH qRT-PCR ELISA
**Studies on serum and/or nasal secretions**						
22	Asano et al., 2017 31	**Serum** Asthma = 20 Asthma + rhinitis = 22 Asthma + CRSwNP = 13 Asthma + CRSsNP = 10	?	Prospective case-control	E as per JESREC	ELISA
23	Maxfield et al., 2017 32	**Serum** CRSwNP = 33 CRSsNP = 38 CLS = 62	?	Prospective case-control	No eosinophil reference	ELISA
24	Xu et al., 2017 26	**Serum** NE = 48 E = 46	?	Prospective case-control	E if eosinophils> 10% of the inflammatory cell population.	Sandwich ELISA
25	Wu et al., 2021 14	**Nasal secretions** Controlled CRSwNP = 30 Uncontrolled CRSwNP = 46	?	Retrospective cross-sectional	Eosinophil count included in multivariate analysis	ELISA

**Abbreviations:** ?, not reported; AR, allergic rhinitis; CRSwNP, chronic rhinosinusitis with nasal polyposis; CLS, controls; E, eosinophilic CRSwNP; ECP, eosinophil cationic protein; ES, ethmoid sinus; IHC, Immunohistochemistry; IHC, immunohistochemistry; ISH, in situ hybrydization analysis; IT, inferior turbinate; L-K, Lund-Kennedy score; L-M, Lund-Mackay score; LW, lateral nasal wall; MT, middle turbinate; NE, noneosinophilic CRSwNP; NERD, nonsteroidal anti-inflammatory drug exacerbated respiratory disease; NP, nasal polyp; qRT-PCR, quantitative real time PCR; RT-PCR, reverse transcription polymerase chain reaction; S, septum; SD, standard deviation; UP, uncinate process.

## Results

The total number of records identified through a database search ( *n* = 229) was reduced to 24 after removing following the procedure presented in Fig. 1 .

Most of the studies were prospective studies on NP samples taken intraoperatively during functional endoscopic sinus surgery (for CRSwNP populations) and septoplasty (for controls), with considerable heterogeneity regarding the anatomical origin of the latter (inferior turbinate, middle turbinate, UP, ethmoid sinus). Almost all research groups have stored the tissue samples at -80°C immediately after collection until the time of protein and mRNA extraction. 9 10 11 12 13 14 15 16 17 18 One study used deparaffinized, rehydrated, and centrifuged samples to acquire a supernatant for measurements. 19 Most of the literature refers to tissue homogenates, where mechanical microdisruptions of fresh tissue permeabilized the cell membranes to extract mRNA and proteins for quantification. Only 6 of the studies conducted a localization analysis. 20 21 22 23 24 25 The majority used reverse-transcription polymerase chain reaction (RT-PCR) for mRNA expression measurements and enzyme-linked immunosorbent assay (ELISA) for protein quantification ( Table 2 ).

Heterogenous criteria have been applied for the diagnosis of E-CRSwNP; the most common one was eosinophils comprising > 10% of the inflammatory cell population. 10 11 23 26 Other researchers used the absolute count of > 7 eosinophils/high-powered field (HPF, 400 × ) 27 or > 10 eosinophils/HPF. 12 The Japan epidemiological survey of refractory eosinophilic CRS (JESREC) classification which includes peripheral blood and radiological findings is also widely accepted. 13 25 Shiono et al. 21 used the following criteria: blood eosinophilia (> 6% of total white blood cells), presence of soft tissue density in the olfactory cleft, and the posterior ethmoid sinus on computed tomography (CT).

Bronchial asthma and the use of glycocorticosteroids for at least 4 weeks before surgery was almost unanimously accepted as sources of bias ( Table 1 ). Regarding corticosteroids, most of the authors excluded patients with history of local as well as systemic use, 10 11 12 14 15 17 18 19 22 23 26 28 29 while others excluded only patients with history of systemic use. 9 13 16 20 25 30 31 Smoking 12 13 14 15 16 17 20 29 31 33 and allergic fungal rhinosinusitis 10 11 13 14 15 19 23 26 were considered as possible sources of bias by several studies. Antihistamines 10 23 and previous sinus surgery 9 16 30 were noted as potential bias in only a few studies. In the study by Wang et al., 22 tissue periostin expressions were found to be significantly upregulated in high-IL-5 compared with low-IL-5 CRSwNP patients, while they did not differ significantly between low-IL-5 CRSwNP and CRSsNP patients. 22

Patient selection concerns were raised for 9 studies ( Tables 1 3 ). Unclear risk of bias concerns regarding the index text were raised for 10 studies, solely comparing NP with control MS tissues. 9 11 12 13 14 15 17 19 20 22 27 28 The majority of studies (n = 18) provided data on E-CRSwNP diagnosis or included tissue eosinophil counts in a multivariate analysis 10 11 12 13 16 18 19 23 25 26 27 29 and were characterized as “low risk” for bias regarding CRSwNP diagnosis and “low concern” regarding the applicability of the results ( Table 3 ).

**Τable 3:** Summary of the results of the QUADAS-2 tool

Study	RoB				Applicability concerns		
	Patient selection	Index test	Reference standard	Flow and timing	Patient selection	Index test	Reference standard
Stankovic et al., 2008 20	☺	?	☹	☺	☺	☺	☹
Platt et al., 2011 30	☺	☺	☹	☺	☺	☺	☹
Ishida et al., 2012 9	☺	☺	☹	☺	☺	☺	☹
Kim et al., 2015 10	☺	☺	☺	☺	☺	☺	☺
Seshardi et al., 2015 33	☹	☺	☹	☺	☺	☺	☹
Shiono et al., 2015 21	☺	☺	☺	☺	☺	☺	☺
Wang et al., 2015 22	☺	?	☺	☺	☺	☺	☺
Asano et al., 2017 31	☺	☺	☺	☺	☺	☺	☺
Gevaert et al., 2017 28	☺	?	☺	☺	☺	☺	☺
Kim et al., 2017 23	☺	☺	☺	☺	☺	☺	☺
Maxfield et al., 2017 32	☹	☺	☹	☺	☺	☺	☹
Xu et al., 2017 26	☹	☺	☺	☺	☺	☺	☺
Xu et al., 2017 11	☹	☺	☺	☺	☺	☺	☺
Wei et al., 2018 27	☹	?	☺	☺	☺	☺	☺
Wu et al., 2018 12	☺	?	☺	☺	☺	☺	☺
Wu et al., 2018 29	☺	?	☺	☺	☺	☺	☺
Nakayama et al., 2019 13	☺	☺	☺	☺	☺	☺	☺
Du et al., 2021 15	☺	?	☹	☺	☺	☺	☹
Wu et al., 2021 14	☹	☺	☺	☺	☺	☺	☺
Yilmaz et al., 2022 19	☹	?	☺	☺	☺	☺	☺
Zhang et al., 2022 16	☺	☺	☺	☺	☺	☺	☺
Danielides et al., 2025 17	☹	?	?	☺	☺	☺	?
Maharani et al., 2025 18	☺	☺	☺	☺	☺	☺	☺
Suzaki et al., 2025 25	☹	?	☺	☺	☺	☺	☺

**Abbreviations:** RoB, risk of bias.

**Notes:** Risk: ☺, low; ☹, high; and ?, unclear.

The results of the included studies are summarized in Table 4 . Few studies have focused on endotype-oriented measurements of periostin. Endotype differentiation regarded tissue eosinophilia, 10 11 13 23 27 dominant biomarker expression, 18 or high/low-IL-5 CRSwNP. 22 Only 5 studies have reported results on periostin levels in non-NP MS from patients with CRSwNP. 18 23 30 33

**Table 4:** Summary of results

Comparison	Study	Result			
		m-RNA	r-RNA	Protein	Gene expression
**CRSwNP (NP) vs. CLS**	Stankovic et al., 2008 20				POSTN; CRSwNP (NP) > CLS, *p* < 0.01
	Platt et al., 2011 30		18S r-RNA; CRSwNP (NP) > CLS, FC = 7.5, *p* < 0.003		
	Ishida et al., 2012 9	CRSwNP (NP) > CLS, *p* < 0.05		CRSwNP (NP) > CLS, *p* < 0.05	
	Seshardi et al., 2015 33	CRSwNP (NP) > CLS, *p* < 0.001			
	Wang et al., 2015 22			high-IL-5 > CLS, *p* = 0.01 low-IL-5 > CLS, *p* = 0.41	
	Gevaert et al., 2017 28			CRSwNP > CLS, *p* < 0.001	
	Kim et al., 2017 23	CRSwNP (NP) > CLS, *p* < 0.0001		CRSwNP (NP) > CLS, *p* < 0.05	
	Wei et al., 2018 27	CRSwNP (NP) > CLS, *p* < 0.001			
	Wu et al., 2018 12			CRSwNP > CLS, FC = 3.95, *p* < 0.01	
	Du et al., 2021 15			CRSwNP > CLS, *p* = 0.001	
	Yilmaz et al., 2022 19			CRSwNP > CLS, *p* = 0.002	
	Danielides et al., 2025 17	CRSwNP (NP) > CLS, *p* < 0.05		CRSwNP (NP) > CLS, *p* < 0.05	
	Maharani et al., 2025 18			CRSwNP (NP) > CLS, *p* < 0.05	
	**CRSwNP (non-NP) vs CLS**	Platt et al., 2011 30		18S r-RNA; CRSwNP(nonNP) vs. CLS, NS		
Seshardi et al., 2015 33		CRSwNP (nonNP) vs. CLS, NS			
Kim et al., 2017 23		CRSwNP (nonNP) > CLS, *p* < 0.05		CRSwNP (nonNP) vs. CLS, NS	
Maharani et al., 2025 18				CRSwNP (nonNP) > CLS, *p* < 0.05	
**CRSwNP (NP) vs CRSwNP (non-NP)**		Platt et al., 2011 30		18S r-RNA CRSwNP (NP) > CRSwNP (non-NP), p < 0.0001		
	Kim et al., 2017 23	CRSwNP (NP) > CRSwNP (non-NP), *p* < 0.001		CRSwNP (NP) > CRSwNP (non-NP), *p* < 0.05	
	Maharani et al., 2025 18			CRSwNP (NP) > CRSwNP (non-NP), *p* = 0.01	
**E vs non-E CRSwNP (NP)**	Kim et al., 2015 10				
	Kim et al., 2017 23	E (NP) > nonE (NP), *p* < 0.01		E (NP) > nonE (NP), *p* < 0.01	
	Xu et al., 2017 11	E (NP) > nonE (NP), *p* < 0.05		E (NP) > nonE (NP), *p* < 0.05	
	Wei et al., 2018 27	E (NP) > nonE (NP), *p* < 0.001		E (NP) > nonE (NP), *p* < 0.001	
	Nakayama et al., 2019 13			E (NP) > nonE (NP), *p* < 0.05	POSTN; E (NP) vs. nonE (NP), NS
	Maharani et al., 2025 18			No correlation with ECP	
	Suzaki et al., 2025 25	E (NP) > nonE (NP), *p* < 0.05		E (NP) > nonE (NP), *p* < 0.01	
**E-CRSwNP (NP) vs CLS**	Kim et al., 2017 23	E (NP) > CLS, *p* < 0.0001			
	Xu et al., 2017 11	E (NP) > CLS, *p* < 0.01		E (NP) > CLS, *p* < 0.05	
	Wu et al., 2018 29			E (NP) > CLS, FC = 3.95, *p* < 0.001	
	Nakayama et al., 2019 13				POSTN; E (NP) > CLS, *p* < 0.001
	Suzaki et al., 2025 25	E (NP) > CLS, *p* < 0.0001		E (NP) > CLS, *p* < 0.0001	
**Non-E-CRSwNP (NP) vs CLS**	Xu et al., 2017 11	NonE vs. CLS, NS		NonE vs. CLS, NS	
	Nakayama et al., 2019 13			NonE vs. CLS, NS	POSTN; NonE vs. CLS, NS
	Suzaki et al., 2025 25	NonE vs. CLS, NS		NonE > CLS, *p* < 0.05	
**CRSwNP (NP) vs NERD (NP)**	Stankovic et al., 2008 20				POSTN; CRSwNP> NERD, *p* = 0.01
	Ishida et al., 2012 9			CRSwNP vs. NERD, NS	
**CRSwNP (NP) vs CRSsNP**	Seshardi et al., 2015 33	CRSwNP (NP) > CRSsNP, *p* < 0.001			
	Wang et al., 2015 22			high-IL-5 > CRSsNP, *p* = 0.01 low-IL-5 vs. CRSsNP, NS	
	Kim et al., 2017 23	CRSwNP (NP) > CRSsNP, *p* < 0.0001		CRSwNP (NP) > CRSsNP, *p* < 0.01	
	Du et al., 2021 15			CRSwNP (NP) vs. CRSsNP, NS	
**CRSwNP (non-NP) vs CRSsNP**	Seshardi et al., 2015 33	CRSwNP (nonNP) vs. CRSsNP, NS			
	Kim et al., 2017 23	CRSwNP (nonNP) vs. CRSsNP, NS		CRSwNP (nonNP) vs. CRSsNP, NS	
	**Serum**	Asano et al., 2017 31 (asthmatic pts)			CRSwNP > CLS, *p* = 0.0002 CRSwNP > CRSsNP, *p* = 0.002 E > nonE, *p* = 0.02	
Maxfield et al., 2017 32				CRSwNP > CLS, *p* = 0.004 CRSwNP > CRSsNP, *p* = 0.004	
Xu et al., 2017 26				Correlation with E, *p* = 0.022	
Yilmaz et al., 2022 19				CRSwNP > CLS, *p* < 0.001	
Danielides et al., 2025 17		CRSwNP vs. CLS, NS		CRSwNP vs. CLS, NS	

**Abbreviations:** CRSsNP, chronic rhinosinusitis without nasal polyposis; CRSwNP, chronic rhinosinusitis with nasal polyposis; CLS, controls; E, eosinophilic; ECP, eosinophil cationic protein; FC, fold change; mRNA, messenger ribonucleic acid; NERD, nonsteroidal anti-inflammatory drug exacerbated respiratory disease; nonE, noneosinophilic; non-NP, nonpolypoidal mucosal samples; NS, nonsignificant; POSTN, periostin gene; rRNA, ribosomal ribonucleic acid; vs, versus.

Localization analyses in Asian populations have described 3 patterns of periostin expression in NP, a “diffuse” throughout the lamina propria, a “superficial” regarding the subepithelial layers between the basement membrane and the nasal glands and a “mixed” one. The “diffuse” pattern was correlated to comorbid asthma 21 and/or E-CRSwNP. 21 24 Stankovic et al. 20 on the other hand, have observed this pattern only in their study on a Caucasian population.

Secondary outcomes verified a strong and significant correlation between mRNA transcription and protein expression levels in NP and control tissue samples, 12 29 34 despite the fact that mRNA and protein measurements refer to different parts of the mucosa ( Table 4 ). Periostin protein has been mainly localized in the subepithelial regions 22 and basement membrane, 25 while its transcripts have been mainly observed in the epithelium of NP tissue. 22 25 Following these findings, Wang et al. 22 concluded that epithelial cells are probably the main cells producing periostin, which is then deposited into the subepithelial region of a mature NP.

A strong and significant correlation has been also shown between different ELISA methods of measuring serum periostin. 35 However, some kits have been consistently attributing higher levels than others, as a result of two factors. First, the lack of standardization of the periostin laboratory samples processing and assay workflow for either electrochemiluminescence or ELISA methods. 36 Second, the fact that periostin can appear in multiple isoforms at proportions that, according to studies on asthmatic populations, may differ between tissues and disease states. 36 Also, ELISA platforms present differences in monoclonal antibodies' affinities for periostin isoforms and, consequently, different isoform mixtures may attribute substantially different numerical results. 31

On the other hand, serum periostin expression does not necessarily correlate with local mucosal expression. In the study of Wang et al. 22 patients with high mucosal IL-5 expression demonstrated significantly higher mucosal periostin levels than patients with low IL-5 expression, whereas they did not differ in their serum periostin concentrations.

Quantitative synthesis of the studies' results is impeded for two reasons. First, the aforementioned discrepancies between different laboratory kits prohibit numerical comparisons of periostin levels resulting from different platforms. 36 Second, the included studies refer to populations with substantial or unknown heterogeneity regarding high-risk bias factors, such as disease severity, comorbidities, and endotypes, 8 with the eosinophilic/noneosinophilic endotyping additionally being subject to different definition criteria. Qualitative synthesis, however, attributed the results presented in Table 4 .

Regarding endotypes, periostin upregulation in terms of both protein and mRNA only refer to NP tissue samples from patients with E-CRSwNP. 10 11 13 23 25 27 29 This protein's levels and mRNA levels in NP tissue samples from patients with nonE-CRSwNP were significantly lower than E-CRSwNP samples, 10 11 13 23 25 27 and similar to controls. 11 13

- The limited literature seems to agree that periostin upregulation refers to mature NP rather than the adjacent non-polypoidal mucosa.
- The differences between mature NP tissue samples and nonpolypoidal MS collected from adjacent anatomical sites within the same CRSwNP patients were only assessed by 3 studies. 18 23 30 Both periostin protein 23 25 and mRNA levels 23 have been reported as significantly higher in NP than adjacent ÙP samples. In the study by Platt et al., 30 18S r-RNA periostin levels are significantly higher in NP than adjacent MS from the septum, inferior turbinate, middle turbinate, and lateral nasal wall (overlying the lacrimal sac). On the other hand, Maharani et al. 18 reported that periostin protein levels significantly correlated with eosinophils cationic protein levels in UP samples of patients with CRSwNP, but not in their NP samples.
- In the few studies comparing MS and controls, controversial results have been reported. Two studies have concluded that UP samples collected from CRSwNP patients are similar to normal controls in terms of periostin m-RNA, 33 while one study reported significantly higher periostin m-RNA levels in UP. 23 The MS from septum, inferior turbinate, middle turbinate, and the area overlying the lacrimal sac did not differ from controls in one study of periostin r-RNA 30 levels. Finally, regarding periostin protein levels, nonpolypoidal UP samples from CRSwNP patients were found to be similar to controls in one study 23 and significantly higher than controls in another. 25
- It is also noteworthy that MS collected from CRSwNP patients were found to be comparable to samples from CRSsNP in terms of both m-RNA 23 33 and protein 23 periostin levels.

The differences in the synthesis and characteristics of the study populations, as well as in the origin of the samples, the methods used to measure periostin levels, and the criteria applied to diagnose E-CRSwNP, may all implement cross-studies bias ( Tables 1 2 3 4 ). Publication bias may also apply, since studies reporting nonsignificant results often remain unpublished.

Multivariate regression analysis (adjusted for asthma, sex, smoking, NERD, oral/nasal steroids' use, and number of previous sinus surgeries) demonstrated that the presence of NP was an independent predictor of higher serum periostin levels. 32 On the other hand, the clinical validity of periostin as a biomarker for the differential diagnosis between non-E/E-CRSwNP has been questioned, 26 unless used as part of combinatorial biomarker algorithms. 10 26 Similarly, although periostin levels in NP samples 27 and serum 24 have been associated with polyp recurrence after endoscopic surgery, its actual clinical validity as a recurrence biomarker is rather low, both in serum 24 and nasal secretion samples. 14

## Discussion

Results are summarized in Table 4 .

The literature is currently lacking data on periostin levels in the nonpolypoidal sites of the CRSwNP mucosa, thus raising questions on whether it actually contributes to the pathogenesis or is eventually found as a by-product of polyp formation. According to the available studies, periostin upregulation in tissue samples applies to mature eosinophilic NP. Comparisons between NP and nonpolypoidal MS of patients with CRSwNP attributed significantly higher protein, mRNA and 18SrRNA periostin levels in the former. 18 23 30

Comparisons between nonpolypoidal mucosa samples of CRSwNP patients and controls have attributed controversial results, being in some studies comparable in terms of periostin protein, 23 mRNA, 33 and rRNA expression 30 or, in other studies, significantly higher than controls in terms of periostin m-RNA 23 and protein 18 levels. No significant regional variation in periostin gene, 18SrRNA, or protein expression was measured between nonpolypoidal mucosa of the septum, inferior turbinate, middle turbinate, ethmoid labyrinth, or lateral nasal wall in either CRSwNP patients or control subjects. 20 30

Endotype-oriented measurements of periostin levels are currently lacking. Eosinophilic NP (eNP) samples exhibited significantly higher mRNA and protein levels than non-eosinophilic in most studies, 10 11 13 23 25 27 with similar tissue goblet cell hyperplasia and collagen deposition. 27 A significant correlation between the number of eosinophils and the mRNA or protein expression levels of periostin in NP has been advocated by most studies, 19 37 although controversial results have also been reported. 18

The main sources of periostin deposited in eNP are mast cells 23 and nasal epithelial cells. 22 23 25 27 Its contribution to persistent and progressive eosinophilic inflammation and NP formation has been advocated with regard to three aspects. 11 22 38 First, the positive association between periostin protein immunoreactivity and basement membrane thickness, as well as goblet cell hyperplasia. 27 In vitro studies suggested that periostin may stimulate NP-derived fibroblasts to produce vascular endothelial growth factor (VEGF), thus increasing microvascular permeability and local angiogenesis, 11 21 39 as well as eotaxin-2 and Regulated on Activation Normal T-Cell Expressed and Secreted (RANTES), thus further triggering inflammation. 11

Second, the speculation that periostin may contribute to eosinophil localization in NP, can be based on its adherence to IL-5-stimulated eosinophils in vitro. 22 40 Periostin is, however, unlikely to contribute significantly to the recruitment of eosinophils into NPs, since it has not been correlated with eotaxin or tissue eosinophil cationic protein expression or tissue or blood eosinophil numbers. 22 25

A third possible pathway of contribution to E-CRSwNP can be identified through the positive association between periostin and IL-5. Periostin has been significantly correlated with IL-4, 5, and 13 expression in NP samples, 22 23 25 27 33 41 while paired comparisons between NP and nonpolypoidal UP samples of the same patients revealed highly significant differences only for IL-5 and periostin. 42 Therefore, one could assume that although up-regulation of periostin during type-2 inflammation is substantially dependent on IL-4 and 13 signaling, 22 23 25 27 33 43 the progression toward a mature polyp out of the inflamed mucosa seems to require additional pathogenetic factors that may, among others, result in the persistence of high periostin and IL-5 levels. 23 25 27 In a limited number of studies on NP, nasal secretions, or serum, high periostin levels have been associated with other molecules as well, such as 15-LOX-1, 37 thymic stromal lymphopoietin (TSLP), 27 matrix metalloproteinase-3 (MMP-3), 15 tissue inhibitor of metalloproteinases-2 (TIMP-2), 15 cystatin, 14 and IgG4, 44 but the relevant underlying mechanisms remain obscure

Little is known about the mechanisms regulating the systematic or local periostin production in CRSwNP patients and controls. This may explain the heterogeneity of the patient selection bias investigated by the included studies ( Tables 1 3 ). Only 2 studies 9 15 state to have interpreted periostin measurements without knowledge of the clinical diagnosis and origin of the specimens, but this is probably of little importance when referring to rather objective methods, such as ELISA and qRT-PCR (quantitative real time PCR).

## Final Comments

The present study has approached the subject with special consideration to the origin of the specimens and the CRSwNP endotype, highlighting two important results. First, periostin upregulation in NP is highly associated with the eosinophilic and high-IL-5 CRSwNP inflammatory patterns, while non-e-NP are comparable to controls. Second, upregulation of periostin refers to NP samples, while periostin levels in nonpolypoidal mucosa samples of CRSwNP patients are comparable to controls and CRSsNP patients. Further similarly oriented research is needed to understand the role of periostin upregulation in the pathogenesis or preservation of eNP.
